# Mortality associated with cysticercosis in a historical cohort from Britain

**DOI:** 10.1590/0004-282X-ANP-2021-0001

**Published:** 2022-02-28

**Authors:** Gagandeep Singh, Peter Chiodini, Josemir W. Sander

**Affiliations:** 1 Dayanand Medical College, Department of Neurology, Ludhiana, India. Dayanand Medical College Department of Neurology Ludhiana India; 2 University College London, Hospitals Biomedical Research Centre, Queen Square Institute of Neurology, London, WC1N 3BG, UK. University College London Hospitals Biomedical Research Centre Queen Square Institute of Neurology London UK; 3 Hospital for Tropical Diseases and London School of Hygiene and Tropical Medicine, Department of Clinical Parasitology, London, UK. Hospital for Tropical Diseases London School of Hygiene and Tropical Medicine Department of Clinical Parasitology London UK; 4 Chalfont Centre for Epilepsy, Chalfont St Peter, United Kingdom. Chalfont Centre for Epilepsy Chalfont St Peter United Kingdom; 5 Stichting Epilepsie Instellingen Nederland, Heemstede, Netherlands. Stichting Epilepsie Instellingen Nederland Heemstede Netherlands

**Keywords:** Taenia solium, Epilepsy, Intracranial Hypertension, Survival, Mortality, Taenia solium, Epilepsia, Hipertensão Intracraniana, Sobrevida, Mortalidade

## Abstract

**Background:**

The burden of premature mortality associated with human cysticercosis is largely ignored mainly due to poor record-keeping in *Taenia solium* endemic regions.

**Objective:**

To document mortality and survival characteristics of an historical cohort with cysticercosis.

**Methods:**

The years of onset of symptoms and death untill 1957 were extracted from published reports of a British military cohort (n=450) examined in London in the early twentieth century. Data were entered into a Kaplan Meier survival analysis with the presence (or absence) of clinical manifestations as independent variables, which were then fitted into a Cox proportional hazards model to determine their significance.

**Results:**

Cysticercosis was responsible for 24 (52.2%) of 46 deaths in the first 15 years of follow-up in comparison to 7 (19.4%) of 36 deaths in the 20-40 years of follow-up period. In the univariate and Cox analyses, intracranial hypertension (hazard ratio [HR]: 8.26; CI: 4.71, 14.49), ocular cysticercosis (HR: 6.60; CI: 3.04, 14.33), and mental disorder (HR: 3.98; CI: 2.22, 7.13) but not epilepsy (HR: 0.66; CI: 0.20, 2.18) were associated with mortality. Over half of all deaths in the first 15 years of follow-up were attributed to cysticercosis.

**Conclusions:**

Several deaths occurred early after acquiring cysticercotic infection. Intracranial hypertension, ocular cysticercosis, and mental disorder but not epilepsy were predictors of mortality in this cohort.

## RESUMO

**Antecedentes:**

O fardo da mortalidade prematura associada à cisticercose humana é amplamente ignorado, principalmente devido à má manutenção de registros nas regiões endêmicas de Taenia solium.

**Objetivo:**

Documentar as características de mortalidade e sobrevivência de uma coorte histórica com cisticercose.

**Métodos:**

Os anos de início dos sintomas e morte até 1957 foram extraídos de relatórios publicados de uma coorte militar britânica (n = 450) examinada em Londres no início do século XX. Os dados foram inseridos em uma análise de sobrevida de Kaplan Meier com a presença (ou ausência) de manifestações clínicas como variáveis ​​independentes, que foram então ajustadas em um modelo de risco proporcional de Cox para determinar sua significância.

**Resultados:**

A cisticercose foi responsável por 24 (52,2%) de 46 óbitos nos primeiros 15 anos de seguimento em comparação com 7 (19,4%) de 36 óbitos nos 20-40 anos de seguimento. Nas análises univariadas e de Cox, hipertensão intracraniana (razão de risco [HR]: 8,26; IC: 4,71, 14,49), cisticercose ocular (HR: 6,60; IC: 3,04, 14,33) e transtorno mental (HR: 3,98; IC: 2,22 , 7,13), mas não epilepsia (HR: 0,66; IC: 0,20, 2,18) foram associados com mortalidade. Mais da metade de todas as mortes nos primeiros 15 anos de acompanhamento foram atribuídas à cisticercose.

**Conclusões:**

Várias mortes ocorreram logo após a aquisição da infecção cisticercótica. Hipertensão intracraniana, cisticercose ocular e transtorno mental, mas não epilepsia, foram preditores de mortalidade nesta coorte.

## INTRODUCTION

Cysticercosis, denoting human infestation with the larval stage of the pork tapeworm *Taenia solium*, is responsible for serious neurological morbidity in many parts of the world^1^^,^^2^. Neurological involvement, *i.e*., neurocysticercosis is often characterized by seizures, intracranial hypertension, dementia, and focal neurological deficits. Considering the severe manifestations, it is unsurprising that the disorder is associated with increased premature mortality. A recent systematic analysis of cysticercosis deaths in the United States (US) between 1990 and 2002 found that the mean age at death in people with cysticercosis was 41 years^3^. However, there is little information on mortality associated with cysticercosis outside the US, mainly due to ineffective public health systems and lack of surveillance systems for life events^3^^-^^6^. 

A large cohort of people with cysticercosis was systematically followed-up in Britain in the early twentieth century^7^^-^^12^. India was a British colony before it became independent in 1947, and British military personnel were frequently deployed in India before 1947. They and their families remained in India for varying periods of time, usually up to three years. Cysticercosis was present in Britain until it was eradicated, with only few reports in the eighteenth century. Hence, it is safe to assume that British military personnel acquired cysticercosis while serving in India, where it remained mostly undiagnosed. Upon their return, they were examined at the Queen Alexandra Military Hospital in London for neurological symptoms including seizures. The hospital specialized in the treatment of non-combat related disorders. After diagnosis, the returnees were retired from military service but were maintained on follow-up until 1957. The cohort was thus comprised of people diagnosed between 1921 and 1957. 

We performed a secondary analysis of data contained in various published reports on this cohort from the 1920s to 1961. Here we report survival rates and their relationship with various clinical presentations in this historical cohort. 

## METHODS

### Cohort composition

This cohort consisted mainly of members of the British army and their families who had previously served in India and had contracted cysticercosis there. They were identified between 1921 and 1957 and were followed-up by army medics at Queen Alexandra Military Hospital, London^13^^,^^14^. The earliest report of five cases dates back to 1930^7^ but the number increased to 71 cases in 1934^9^, 284 in 1944,^11^ and to 450 in 1957^12^. Deployment of military personnel to India ceased in 1947 and the last case was diagnosed in 1949. In response to the initial reports, the Army supported continued scrutiny of the cases and longitudinal data were systematically collected. The final report was published in 1961 and contained data up to August 1957^12^. 

### Clinical presentations

The final report contained the findings of the autopsy of 55 of the subjects who died before August 1957^12^. The report included the forms of presentation coded as epilepsy, focal neurological deficit, intracranial hypertension, mental disorder, ocular cysticercosis, subcutaneous involvement, lingual cysticercosis, muscular disorder, and acute febrile illness, as well as the year of symptom-onset and death. The report also categorized the causes of death into those due to cysticercosis and those due to other causes. Death was attributed to cysticercosis if the immediate or underlying cause was reported as related to epilepsy (status epilepticus, drowning, phenobarbital overdose, and suicide), intracranial hypertension, or cysticercotic meningitis. We used the reported year of symptom onset and year of death in those who died^12^. In those who remained asymptomatic, the year of exposure was assumed from the mid-point of their deployment in India. The original reports also provided data on asymptomatic cases, which were also included in examination of their association with survival^7^^-^^12^.

### Statistical analysis

Data were entered into a Kaplan Meier survival analysis, and subjects were considered at risk of dying from the year of symptom onset. They exited at either death or censoring, whichever occurred earlier. Censoring was at the time of the last follow-up (August 1957). The association between the presence or absence of each category of clinical manifestations was examined in univariate analyses. The presence or absence of manifestations was then fitted as a covariate in a Cox proportional hazard model to estimate exponential individual coefficients and hazard ratios. The coefficients represent the hazard ratio for a unit change in the covariate. Stata version 15.1 (StataCorp, TX, USA) was used for analysis.

## RESULTS

The final report provided details of 450 cases up to August 1957^12^. The diagnosis of cysticercosis in the cohort was established by biopsy of subcutaneous nodules in 108 (24%), by radiology of the limbs and skull in 313 (70%), by autopsy or surgery in 22 (5%), and by ocular cysticercosis in 2^12^. 

Total follow-up accounted for 9713 person-years starting in 1891, the year of symptom-onset in the first subject. Three-hundred-fifty-six (79%) individuals were alive in 1957. Survival at one year was 99% (95% Confidence Intervals [CI]: 98%, 100%), at five years, 95% (CI: 93%, 97%) and at 10 years, 92% (CI: 89%, 94%). Of the 94 (21%) deaths, 47 (50%) were attributed to cysticercosis. When only deaths attributed to cysticercosis were considered, the cause-specific survival at one year was 99% (CI: 98, 100), at five years 96% (CI: 93%, 97%) and at 10 years, 93% (CI: 91%, 95%). The median overall survival was 23 years (range: 1-59 years). Overall mortality rate was 9.7 (CI: 7.6, 11.5) per 1000 person-years. Considering cysticercosis-related deaths only, the estimated mortality rate was 4.6 (CI: 3.5, 6.2) per 1000 person-years.

Seven (47.6%) of 18 deaths in the first 5 years of follow-up, 9 (56.3%) of 16 deaths in the next five years, and 8 (66.7%) of 12 deaths in the 11-15 years follow-up period were attributed to cysticercosis. In comparison, the proportional mortality attributed to cysticercosis was comparatively lower from 20 years onwards (21-25 years: 2 [20%] out of 10; 26-30 years: 4 [30.8%] out of 13; 31-35 years: 1 (10%) out of 10, and 36-40 years: none out of 3). Overall, 27 (81.8%) of the cysticercosis-related deaths and 49 (59.8%) of 91 all-cause deaths occurred in the first 15 years of follow-up. 

### Survivor function and clinical presentations

The clinical features of the cohort are shown in Table 1. Epilepsy was the commonest presentation, with 413 (91.8%) affected people. Subcutaneous nodules were detected in 243 (54%) subjects. In all, only 15 (3.3%) subjects remained asymptomatic throughout the observation period.

**Table 1.** Clinical characteristics of the cohort.

Clinical presentation	Number (%) N=450
Asymptomatic	15 (3.3%)
Epilepsy	413 (91.8%)
Focal nervous lesion	13 (2.9%)
Intracranial hypertension	29 (6.4%)
Mental disorder	38 (8.4%)
Ocular cysticercosis	9 (2.0%)
Subcuticular nodules	243 (54.0%)
Lingual cysticercosis	8 (1.8%)
Muscular pain	15 (3.3%)
Acute febrile illness	6 (1.3%)

*Subjects could have more than one clinical presentation

**Figure 1.**
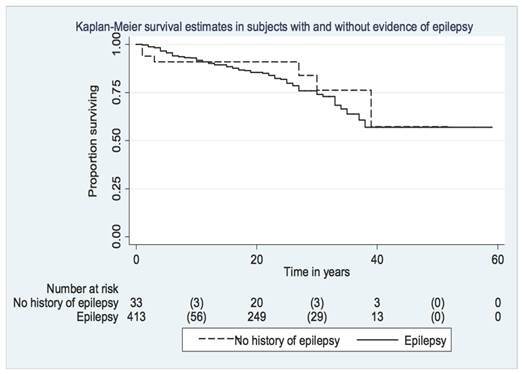
Kaplan Meier survival curves in individuals with and without epilepsy in the British military cohort.

**Figure 2.**
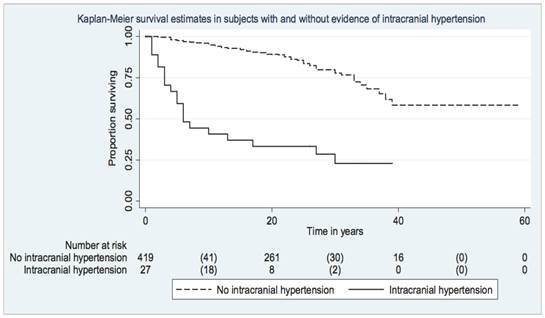
Kaplan Meier survival curves in individuals with and without intracranial hypertension in the British military cohort.

The characteristics of survivors (Table 2) were similar in people with and without (asymptomatic subjects with cysticercosis) symptoms (P=0.42), those with and without epilepsy (P=0.54) (Figure 1), those with and without focal neurological deficits (P=0.27), and those with and without subcutaneous infestation (P=0.45). Conversely, survival was lower in those with intracranial hypertension (median survival: 6 years) (P=0.00001) (Figure 2), mental disorder (median survival: 13 years) (P=0.00001), ocular cysticercosis (median survival: 9 years) (P=0.00001), and those with a febrile illness (P=0.004), each compared with those without the same presentation. Mortality rates associated with cysticercosis were generally lower in subjects with epilepsy (5/1000 person-years; CI: 3-6/1000 person-years) and subcutaneous cysticercosis (5/1000 person-years; CI: 4-8/1000 person-years), but it was higher in subjects with lingual involvement (14/1000 person-years; CI: 4-51/1000 person-years), mental disorder (20/1000 person-years; CI: 12-34/1000 person-years), febrile illness (31/1000 person-years; CI: 8-125/1000 person-years), ocular cysticercosis (34/1000 person-years; CI: 14-53/1000 person-years), and intracranial hypertension (47/1000 person-years; CI: 29-76/1000 person-years).

**Table 2.** Overall and cause-specific mortality associated with different clinical manifestations.

Clinical presentation		N	Overall mortality; O/E	Overall mortality rate (per 1000 person-years)	Cause-specific mortality; O/E	Cause-specific mortality rate (per 1000 person-years)	Median survival (95%CI) (years)	Significance (overall mortality) P	Significance (cause-specific mortality) P
Asymptomatic		15	3/5	7 (2,22)	45/43	NA	30 (17-59)	0.41	0.19
Symptomatic		435	88/86	NA	0/2	5 (3, 6)			
Epilepsy	Yes	413	85/83	9 (8,12)	42/4	5 (3, 6)	23 (1-59)	0.53	0.87
	No	37	6/7		3/3	4 (1, 12)			
Focal nervous lesion	Yes	13	4/2	15 (6,39)	0/1	5 (4, 6)	22 (10-31)	0.27	0.24
	No	437	87/89		45/44				
Intracranial hypertension	Yes	29	20/4	55 (36, 86)	17/2	47 (29, 76)	6 (1-39)	0.00001	0.00001
	No	421	71/87		28/43	3 (2, 4)			
Mental disorder	Yes	38	17/6	26 (16, 42)	13/3	20 (12, 34)	13 (1-49)	0.00001	0.00001
	No	412	74/85		32/42	4 (2, 5)			
Ocular cysticercosis	Yes	9	8/1	55 (28, 110)	5/1	34 (14, 53)	9 (3-49)	0.00001	0.00001
	No	441	83/90		40/44	4 (3, 6)			
Lingual cysticercosis	Yes	8	4/1	28 (11, 76)	2/1	14 (4, 57)	15 (4-31)	0.16	0.13
	No	442	87/90		43/44	4 (3, 6)			
Acute febrile illness	Yes	4	3/1	47 (15, 145)	2/0	31 (8, 125)	15 (1-33)	0.004	0.002
	No	446	88/90		43/44	4 (3, 6)			
Subcutaneous nodules	Yes	243	45/49	9 (6, 11)	21/25	5 (4, 8)	22 (2-59)	0.44	0.29
	No	207	46/42		24/20	4 (3, 6)			
Muscular pain	Yes	15	1/3	3 (1, 20)	0/2	-	5 (4-6)	0.18	0.19
	No	435	90/88		45/43				

O/E (Observed/Expected mortality ratio): This is the ratio of the observed mortality associated with an attribute to the mortality predicted from the proportion of the cohort population with the attribute.

When clinical variables were fitted in a Cox proportional hazards model to test overall survival, the model was significant (P=0.00001) with intracranial hypertension (hazard ratio [HR]: 8.26; CI: 4.71, 14.49), ocular cysticercosis (HR: 6.60; CI: 3.04, 14.33), and mental disorder (HR: 3.98; CI: 2.22, 7.13) being significant predictors of survival (Table 3). The three clinical variables remained significant predictors of survival in the model to test cause (cysticercosis)-specific survival (overall P of the model = 0.00001). 

**Table 3.** Hazard ratios for overall and cause-specific survival outcomes associated with different clinical manifestations.

**Clinical presentation**	Overall survival Hazard Ratio (95% CI); P	Cause-specific (cysticercosis-related) survival Hazard Ratio (95% CI); P
Asymptomatic Vs. Symptomatic Individuals	0.610 (0.114-3.259); 0.563	0.000 (0.0 -0.0); 1.000
Epilepsy Vs. No epilepsy	0.662 (0.201-2.179); 0.497	0.294 (0.083-1.045); 0.058
Focal nervous lesion	2.468 (0.840-7.254); 0.100	0.000 (0.0-0.0); 1.000
Intracranial hypertension	8.260 (4.710-14.486); 0.0001	19.621 (9.633-39.965); 0.000
Mental disorder	3.978 (2.221-7.125); 0.0001	7.954 (3.823-16.549); 0.000
Ocular cysticercosis	6.599 (3.040-14.325); 0.0001	5.826 (2.119-16.018); 0.001
Subcuticular cysticercosis	0.772 (0.504-1.182); 0.233	0.700 (0.383-1.279); 0.246
Lingual cysticercosis	1.474 (0.496-4.377); 0.485	0.961 (0.210-4.390); 0.959
Muscular pain	0.202 (0.025-1.604); 0.130	0.000 (0.0 -0.0); 1.000
Acute febrile illness	0.849 (0.246-2.934); 0.796	0.547 (0.113-2.633); 0.452

CI: confidence interval.

## DISCUSSION

Evidence derived from this cohort has had a profound influence on the current understanding of cysticercosis^13^^,^^14^. Our analysis of mortality and survival provides further insights, as cysticercosis-related mortality is a major public health issue largely neglected partly due to the constraints of poor record-keeping in endemic areas^3^^-^^6^. Despite the limitation of the absence of data on the age characteristics of the cohort, our findings suggest that cysticercosis substantially contributed to premature mortality. This is because cysticercosis usually occurs in young people and most deaths attributed to cysticercosis occurred in the first 15 years of exposure in this historical cohort^15^^-^^20^. Only very recently has the premature mortality associated with infestation been confirmed by an analysis of cysticercosis-related deaths in the USA, which estimated a mean age at death of 40.5 years^3^. 

How does the mortality estimate of 4.6/1000 person-years in our cohort compare with current data? A Brazilian report estimated a crude mortality rate of 0.88-2.51/million people in 1995-2004^6^. In comparison, analysis of the nationwide inpatient sample from the USA produced an estimate of 0.1 deaths/million population^15^. When death certificates from the National Centre for Health Statistics were the source, mortality estimates for the period 1990-2002 amounted to 0.06/million population^3^. 

Careful documentation of presentations and year of symptom-onset and death in the whole cohort provided an opportunity to assess the relationship between various clinical presentations and mortality. In the Cox model, intracranial hypertension, mental disorder, and ocular cysticercosis, but not epilepsy, were associated with reduced survival (Table 3). Intracranial hypertension is a severe manifestation of cysticercosis and might occur as a result of non-obstructive hydrocephalus associated with basal sub-arachnoid cysticercosis, obstructive hydrocephalus due to intraventricular cysticercosis, giant solitary cysticerci, or multiple parenchymal cysticerci with massive cerebral edema^16^^-^^20^. Two conditions, cysticercotic encephalitis and disseminated cysticercosis, characterized by massive brain infection and intracranial hypertension^18^^,^^19^ are still associated with high case fatality rates. Obstructive hydrocephalus could have contributed to death in seven subjects who underwent neurosurgical treatment, as five of them died soon after probably due to the procedure^12^. A Polish report from the 1960s suggested that nearly two thirds of those with hydrocephalus resulting from basal arachnoidal cysticercosis died from the disease^20^. The only available surgical treatment for obstructive hydrocephalus at the time was ventriculostomy. Shunt procedures were implemented several decades later and led to a decline in mortality associated with hydrocephalus^21^. Likewise, the application of medical treatment in the form of repeated courses of albendazole signaled a major breakthrough in the management of subarachnoid cysticercosis with intracranial hypertension^22^. Besides intracranial hypertension, both mental disorders and ocular cysticercosis were associated with reduced survival. Mental disorders, described in many early reports, are also associated with heavy cerebral infection^12^^,^^18^^,^^23^^,^^24^. Presumably, many subjects in the cohort had heavy infection loads, which is supported by the fact that more than half of the subjects had subcutaneous nodules, a surrogate for a heavy cyst burden^12^. 

 The lack of an association of mortality with epilepsy is difficult to explain, especially in view of the well-established association between epilepsy and premature mortality (Tables 2 and 3; Figure 1) ^25^. An original report analyzed the causes of death in a representative sample and found status epilepticus as the immediate cause of death in 18 cases (43%)^12^. A possible explanation arises from the small proportion of patients without epilepsy (n=37 of 450; 8%; Appendix, Table 1), which could inadvertently inflate the number of deaths in the subgroup without epilepsy in a cohort with severe cysticercosis. Alternatively, the occurrence of seizures might have led to earlier diagnosis (and thus better prognosis) by physicians familiar with epilepsy as a manifestation of cysticercosis^7^^-^^9^. A parallel can be drawn from studies of cerebral glioma, in which the occurrence of seizures is an independent predictor of improved survival^26^. 

Our findings must be interpreted in light of the prevailing standards of care at the time. One discernible difference between the historical and modern series relates to the available diagnostic tools for neurocysticercosis. In this cohort, limb and skull x-rays and biopsies of subcutaneous nodules were the main diagnostic methods and were perhaps only able to diagnose the more severe infestations^7^^-^^12^. Currently, neuroimaging is almost always used to establish the diagnosis of neurocysticercosis and this has led to the recognition of milder forms of infestation with solitary (or 1-2 cysts) parenchymal cysticercosis, a condition with an excellent prognosis^27^^,^^28^. Some of the medications that are the standard of care today, such as the anthelminthic agents albendazole and praziquantel, and intravenous agents used to treat status epilepticus were not available in the early twentieth century^29^^-^^33^. Our study was also limited by the absence of data on the age of individual subjects, which precluded the estimation of age-adjusted mortality rates. The findings of the association between intracranial hypertension, mental disorders, and ocular cysticercosis and reduced survival are noteworthy and currently relevant. We believe that our analysis is not only of historical interest, as it also provides a useful estimate of mortality and survival in people with cysticercosis. 
